# Construction of Novel Aspartokinase Mutant A380I and Its Characterization by Molecular Dynamics Simulation

**DOI:** 10.3390/molecules23123379

**Published:** 2018-12-19

**Authors:** Caijing Han, Li Fang, Chunlei Liu, Yunna Gao, Weihong Min

**Affiliations:** 1 College of Food Science and Engineering, Jilin Agricultural University, Changchun 130118, China; hancaijingsmile@163.com (C.H.); fangli1014@126.com (L.F.); liuchunlei0709@jlau.edu.cn (C.L.); gaoyunna3920@163.com (Y.G.); 2 National Engineering Laboratory of Wheat and Corn Deep Processing, Jilin Agricultural University, Changchun 130118, China

**Keywords:** novel monomer, aspartokinase, mutant strains, molecular dynamics simulation, open conformation, inhibitors

## Abstract

In this study, a novel monomer aspartokinase (AK) from *Corynebacterium pekinense* was identified, and its monomer model was constructed. Site 380 was identified by homologous sequencing and monomer model comparison as the key site which was conserved and located around the binding site of the inhibitor Lys. Furthermore, the mutant A380I with enzyme activity 11.32-fold higher than wild type AK (WT-AK), was obtained by site-directed mutagenesis and high throughput screening. In the mutant A380I, the optimal temperature was raised from 26 °C (WT-AK) to 28 °C, the optimal pH remained unchanged at 8.0, and the half-life was prolonged from 4.5 h (WT-AK) to 6.0 h, indicating enhanced thermal stability. The inhibition of A380I was weakened at various inhibitor concentrations and even activated at certain inhibitor concentrations (10 mM of Lys, 5 mM or 10 mM of Lys + Thr, 10 mM of Lys + Met, 5 mM of Lys + Thr + Met). Molecular dynamics simulation results indicated that the occupancy rate of hydrogen bond between A380I and ATP was enhanced, the effect of Lys (inhibitor) on the protein was weakened, and the angle between Ser281-Tyre358 and Asp359-Gly427 was increased after mutation, leading to an open conformation (R-state) that favored the binding of substrate.

## 1. Introduction

Aspartokinase (AK) is the first key allosteric enzyme of aspartase family [1] in the biosynthetic pathway, which catalyzes the reaction between l-aspartic acid and ATP to generate aspartyl phosphoric acid and ADP. It can flexibly control carbon flow and adjust reaction speed in the synthesis pathway, acting as the main rate-limiting enzyme [2]. The enzyme plays an important role in the biosynthesis of lysine, threonine and methionine, and has, hence, attracted much attention [3,4].

Recent research has found that AK from different organisms has different forms of feedback inhibition. For example, the AKI from *Arabidopsis* shows cooperative feedback inhibition by lysine and methionine [5,6], and AK from *thermophilic bacteria* has feedback inhibition only by threonine [7,8]. The three kinds of AK from *Methancoccus* are threonine sensitive [9,10]; AKI and AKII from *Escherichia coli* show feedback inhibition by threonine and methionine [11,12], and AKIII by lysine from the metabolites [10]; AK from *Corynebacterium glutamicum* (CgAK) demonstrates cooperative feedback inhibition by threonine and lysine [13].

With the gradual accumulation of evidence in support of the important role played by AK in plant and microbial metabolism [14,15], researchers have initiated studies on the basic structure of AK [1,13]. At present, the crystal structure of AK is known to mainly exist in the form of homotypic and heterotypic oligomers. AKIII from *E. coli* is a homodimer [16]; AK from *Arabidopsis* is a homooligomer [17]; and AK from *Corynebacterium glutamicum* [13], *thermophilic bacteria* [18] and *Mycobacterium tuberculosis* [17] is a heterotetramer (α_2_β_2_), composed of isomolar α and β subunits. The α-subunit contains a catalytic domain at the N-terminus and regulatory domain at the C-terminus. The β-subunit is the same as the regulatory domain of the α-subunit. A completely new heterodimer (αβ) was identified by analyzing the crystal structure of AK from *cyanobacteria* [19]. In addition, the AK from *Clostridium acetobutylicum* has been found to have a tetrameric catalytic domain conformation by comparing crystal structures [20].

**Figure 1:**
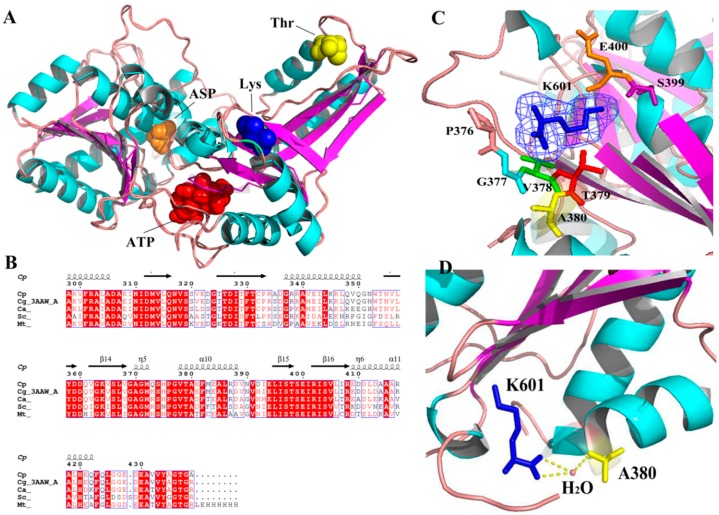
Position of site 380 in the model. (**A**) The novel monomeric model of AK and the docking position of Asp, ATP, Lys and Thr. (**B**) Homologous alignment generated by MEGA6 and modified by ESPript. (**C**) The residue around the binding site of the inhibitor Lys (at site 601 and shown in blue mesh). (**D**) Site 380 with the inhibitor. Blue: inhibitor lysine Yellow: site 380. Site 380 is connected to the inhibitor Lys by a water bridge.

The reports cited above mainly discuss AK in the form of a multimer. However, interestingly, we had identified a novel monomeric AK from *Corynebacterium peking* (CpAK), which was also regulated by the feedback of threonine and lysine (data not shown, in another upcoming article). The homology between A chain of CgAK and the novel monomeric AK was as high as 98% by sequence alignment. Therefore, we have built a model of the novel monomeric AK, based on the A chain of crystal structure (3AAW) of CgAK (Figure 1A) (data not shown, in another upcoming article). Compared with homologous alignment, we found site 380 to be conserved (Figure 1B); the site was located around the binding site of the inhibitor Lys, (Figure 1C) and was connected to the inhibitor Lys by a water bridge (Figure 1D). Therefore, we conjectured that 380 was the key site for regulating feedback inhibition. A mutant A380I was obtained by site-directed saturation mutagenesis and high throughput screening. Molecular dynamics (MD) simulation was used to explore the mechanism of enhancing enzyme activity and relieving feedback inhibition, preliminarily. The effect of Lys (inhibitor) on A380I was weakened; the occupancy rate of the hydrogen bond between A380I and ATP was enhanced, and the conformation was open, which favored the binding of the substrate.

## 2. Results and Discussion

### 2.1. Construction and Purification of Mutant Strains

**Figure 2:**
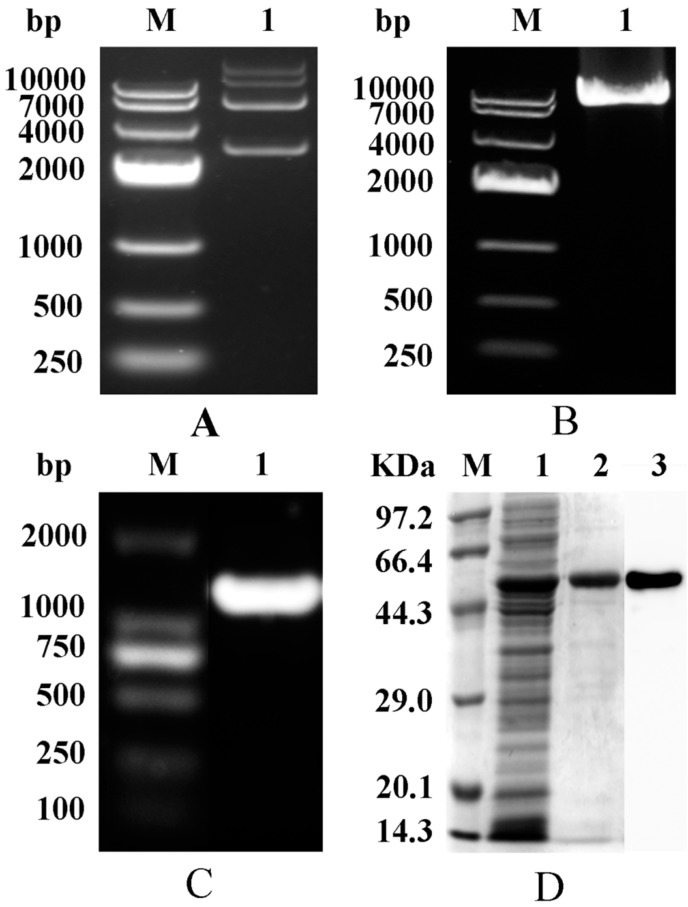
Structure and verification of AK. (**A**) Agarose gel electrophoresis of plasmid (PET-28a-AK). M: DNA marker; 1: WT-AK; (**B**) Agarose gel electrophoresis of the plasmid PCR. M: DNA marker; 1: A380I; (**C**) Agarose gel electrophoresis of bacterial PCR products. M: DNA marker; 1: A380I; (**D**) SDS-PAGE and Western Blot. M: protein marker; 1: crude enzyme sample; 2: purified A380I; 3: Western Blot.

As shown in Figure 2A, the recombinant plasmid was about 7000 bp (pET-28a 5369 bp, AK gene 1266 bp). The band of the PCR product, obtained using the recombinant plasmid as the template and appropriate mutational primers, was also about 7000 bp (Figure 2B), indicating successful mutation. In addition, the band of amplified AK gene, obtained by PCR using the activated bacterial solution as the template and cloned primers as the primers, was at 1266 bp (Figure 2C). The strain was sent for sequencing after verification of mutation by PCR. The mutant strain was induced to express AK enzyme and was purified by Ni Sepharose™ 6 Fast Flow. The enzyme was confirmed by SDS-PAGE and Western blot due to the appearance of a band at 48 kDa (Figure 2D).

### 2.2. Dynamics and Enzymatic Properties

**Figure 3:**
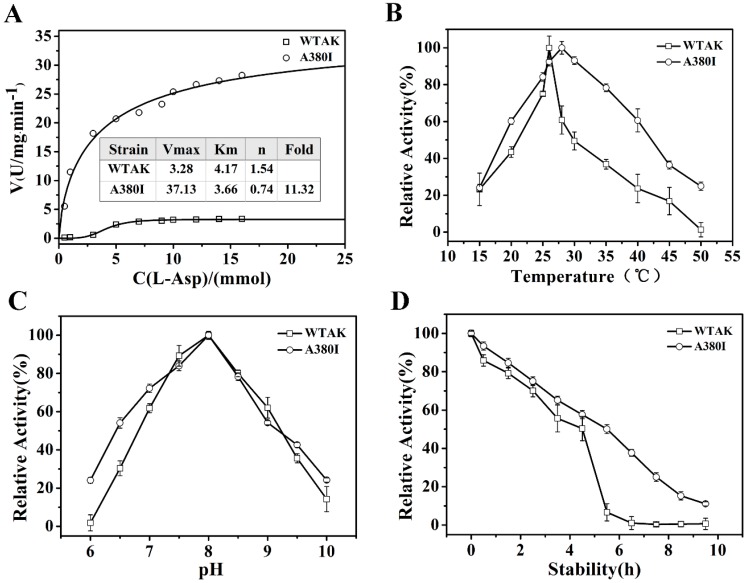
Dynamic analysis and enzymatic properties. (**A**) Dynamic analysis; open circle represents A380I; open square represents WT-AK, the same below. (**B**) The optimal temperature. (**C**) The optimal pH. (**D**) Thermal stability.

The kinetic parameters of wild type AK (WT-AK) and A380I are shown in Figure 3A. The n value represented the Hill coefficient from the Hill equation fit. The n value of WT-AK was 1.54, indicating its positive association with typical allosteric enzymes; that of A380I was 0.74 after mutation. Mutant AK was changed from a positive association to negative synergy. The Km value of WT-AK was 4.17, which reduced to 3.66 after mutation. The reduction in Km value indicated its enhanced affinity to the substrate. After mutation, the maximum reaction rate increased from 3.28 U/mg·min^−1^ to 37.13 U/mg·min^−1^, and catalytic activity was indicated significantly (*p* < 0.05) (11.32-fold higher than WT-AK). The fold represents the ratio of the enzyme activity of the mutant strain A380I to that of the wild type.

As shown in Figure 3B, the optimal temperature of WT-AK increased after mutation (A380I) with values of 26 °C and 28 °C, respectively. The enzyme activity of the mutant was seen to be higher than that of WT-AK when the temperature was either lower than 25 °C or higher than 28 °C, indicating that tolerance temperature had increased in the mutant. The optimal pH of both WT-AK and A380I was 8.0 (Figure 3C). However, when the pH was lower than 7.0, the activity of A380I was higher than that of WT-AK, indicating enhanced acid resistance, which would be conducive for subsequent fermentation [21]. The half-life of AK was extended from 4.5 h to 6 h after mutation, indicating enhanced enzyme stability by mutation (Figure 3D).

According to Table 1, the inhibition of WT-AK activity was positively correlated with the inhibitor concentration. The highest inhibition rate reached 25% for a single inhibitor; the effect was increased to 65% due to the concurrence of two inhibitors, Lys and Thr. The inhibitory mechanism (by the synergistic feedback of Thr and Lys) in the novel AK was similar to that in CgAK [13]. The highest inhibition rate reached 70% by the simultaneous action of three inhibitors. A380I tended to attenuate inhibition: With Lys alone as the inhibitor, the inhibitory effect slowed down with increased concentration of Lys and actually showed activation at 10 mM Lys. The inhibitory effect also weakened due to the concurrence of two inhibitors (Lys + Thr, Lys + Met, and Thr + Met); especially when Lys was included, it showed activation at high concentrations. The inhibitory effect also weakened when three inhibitors existed together. This suggests that site 380 affects the binding of inhibitors to AK.

### 2.3. Analysis of MD Simulation

**Figure 4:**
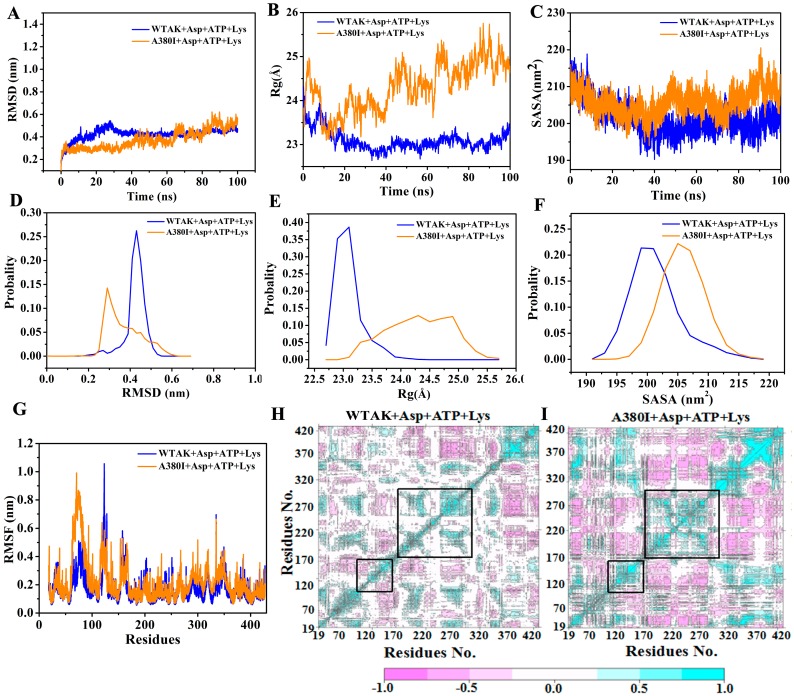
(**A**) RMSD plot during 100 ns MD simulations. Blue represents WT-AK + Asp + ATP + Lys and orange represents A380I + Asp + ATP + Lys, the same below. (**B**) Rg plot. (**C**) SASA plot. (**D**) The probability of RMSD of two complexes (WT-AK + Asp + ATP + Lys and A380I + Asp + ATP + Lys). (**E**) The probability of Rg. (**F**) The probability of SASA. (**G**) RMSF plot. (**H**) Domain cross correlation analysis performed on all Cα atom pairs of WT-AK with Asp, ATP and Lys. (**I**) Domain cross correlation analysis performed on all Cα atom pairs of A380I with Asp, ATP and Lys. The positive correlation region marked in the cyan indicated the strong correlated movement of the residue. The negative correlation region marked in the pink indicated anti-correlation movement of the residue. The diagonal was related to the relative height, representing the variance of the residue with itself.

In order to investigate the reasons for enhancing enzyme activity and relieving feedback inhibition after mutation, two complex systems (WT-AK + Asp + ATP + Lys and A380I + Asp + ATP + Lys) were employed for 100 ns MD simulations. The relative stability of WTAK indicated that the simulation was feasible and could be used for subsequent analysis. RMSD of two complex systems reached equilibrium after the 10 ns simulation (Figure 4A) [22], the values being 0.32 nm and 0.30 nm, respectively. Average fluctuations of the RMSD of the WT-AK + Asp + ATP + Lys and A380I + Asp + ATP + Lys were 0.42 nm and 0.37 nm, respectively. The RMSD value of WT-AK + Asp + ATP + Lys complex system remained mostly at 0.45 nm, whereas the RMSD value of A380I + Asp + ATP + Lys complex system showed significant differences, mostly remaining at 0.3 nm (Figure 4D). The lower RMSD values of A380I indicated enhanced stability of the mutant strain after ligand binding. The radius of gyration (Rg, Figure 4B) represents the overall size of the protein [23]. Rg values of the complex system WT-AK + Asp + ATP + Lys showed that inhibitors (Lys) reduced the flexibility of the protein (remaining mostly 23.0 Å, Figure 4E), whereas Rg values of A380I + Asp + ATP + Lys system showed significant fluctuations (remaining mostly 24.3–25 Å, Figure 4E) during the simulation process, indicating that mutation of alanine (A) into isoleucine (I) near the inhibitor would restore the flexibility and activity of CpAK. Solvent-accessible surface area (SASA, Figure 4C) represents the affinity between proteins and polar solvents [24]. Protein with higher SASA value has stronger affinity for solvents. The SASA value of WT-AK + Asp + ATP + Lys complex system remained mostly 200 nm^2^, whereas the SASA value of A380I + Asp + ATP + Lys complex system showed significant differences, mostly remaining at 205 nm^2^ (Figure 4F). The SASA value of A380I + Asp + ATP + Lys complex system was higher than that of WT-AK + Asp + ATP + Lys complex system, indicating the enhanced affinity of A380I to the solvent.

The effect of the inhibitor on protein SASA might have been eliminated after the mutation in the neighborhood of the inhibitor; hence, it was helpful to catalyze the reaction and increase the enzyme activity. Root mean square fluctuation (RMSF, Figure 4G) could evaluate the flexibility of each residue in the complex systems [25]. Val120–Gly170 and Leu220–Glu270, which surrounded the binding pocket of Asp and ATP, showed significant fluctuations compared to other residues, and the fluctuations increased after mutation (Figure 4G).

To further explain the above observation, covariance matrices of the two complexes (WT-AK + Asp + ATP + Lys and A380I + Asp + ATP + Lys) were tested. The extent of violent motion could be emphasized by the diagonal matrix. As shown in Figure 4H,I, A380I mutant had stronger positive and negative correlation motions than WT-AK. The cyan color increased in the regions of residues Val120–Gly170 and Leu220–Glu270 of A380I, implying that these two regions had mainly positive correlation motion and the result was consistent with the fluctuation in RMSF.

AK from different biosomes is inhibited by the feedback of different metabolites. CgAK was inhibited by the synergistic feedback of threonine and lysine; when CgAK did not bind to the inhibitor, its structure was relatively relaxed (CgAK-R) and may be called an active state [26]. However, its structure causes obvious allosterism to remain in a tense state, such that it is in inactive conformation when Thr and Lys exist together. The specific mechanism may be that the ionic bond between Arg151 and Glu74 prevented the binding of aspartic acid and stabilized the inactive state of CgAK-T. As seen from Table 1, the novel AK was inhibited by the synergistic feedback of threonine and lysine. By MD simulation, we also found that inhibitors (Lys) could reduce the flexibility of the protein (Figure 4B) as well as the fluctuation of residues (Val120–Gly170 and Leu220–Glu270) near the pockets of ATP and Asp (Figure 4G–I). We, therefore, inferred that the novel AK and CgAK had similar inhibitory mechanisms involving binding in the active sites, which were also the pockets of ATP and Asp. We have studied the active sites in detail.

### 2.4. Mutations Affect the Flexibility of Active Site Residues

**Figure 5:**
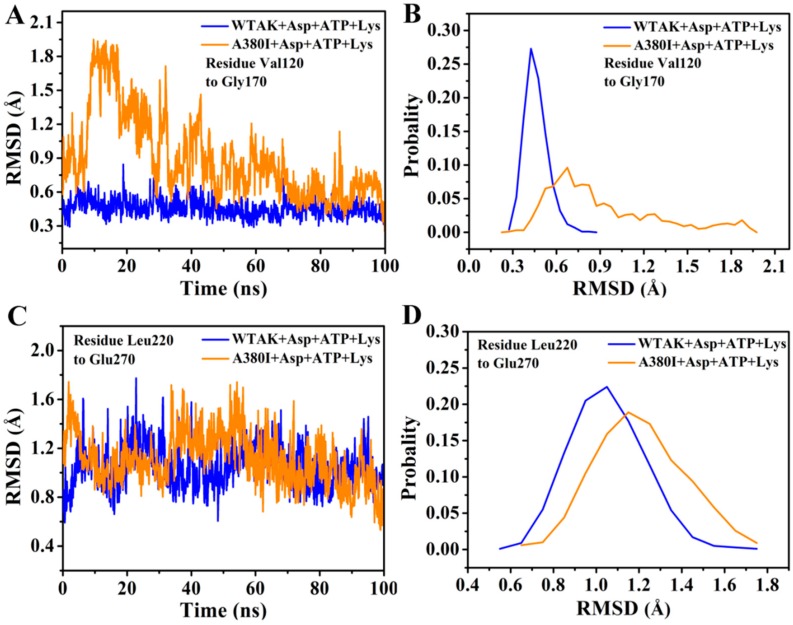
RMSD analysis of two complexes. (**A**) RMSD from residues Val120–Gly170. (**B**) The probability of RMSD from residues Val120–Gly170. (**C**) RMSD from residues Leu220–Glu270. (**D**) The probability of RMSD from residues Leu220–Glu270.

The RMSD values of Val120–Gly170 and Leu220–Glu270 were calculated by 100 ns simulation (Figure 5); they remained approximately 0.4 nm in WT-AK + Asp + ATP + Lys complex system and mostly exceeded 0.9 nm in A380I + Asp + ATP + Lys complex system. It indicated that A380I could increase the RMSD values of Val120–Gly170 of the Asp binding pocket (Figure 5A,B), thereby enhancing the interaction with Asp. This was consistent with Thongekkaew’s report, which demonstrated that the increase in enzyme activity is due to the increase in hydrogen bonding between enzymes and substrates [27].

Similarly, A380I showed increased RMSD of residues Leu220–Glu270 in the ATP binding pocket (Figure 5C,D). We further analyzed the specific interaction between mutant A380I and ATP. From the hydrogen bond occupancy of ATP and CpAK in the two complex systems, Arg203, Ser227 and Lys 228 had strong hydrogen bonding with CpAK (Table 2). The occupancy ratio of the hydrogen bond between ATP and CpAK increased. The occupancy ratio of hydrogen of Arg203 (NH1)-ATP (O) increased from 38.34% to 51.78% in A380I+Asp+ATP+Lys complex system. The results showed that A380I increased the binding of ATP and CpAK, thereby enhancing the activity of CpAK.

**Figure 6:**
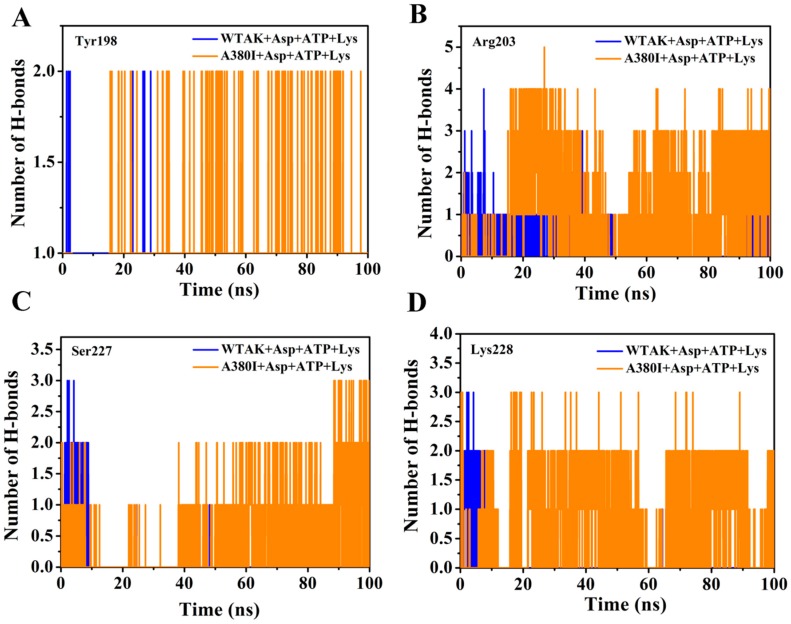
Number of H-bonds between different sites and the ATP of two complex systems (WT-AK + Asp + ATP + Lys and A380I + Asp + ATP + Lys) in 100 ns dynamic simulation. (**A**) Tyr198. (**B**) Arg203. (**C**) Ser227. (**D**) Lys228. WT-AK + Asp + ATP + Lys complex systems show in blue. A380I + Asp + ATP + Lys complex systems show in orange.

The number of H-bonds was acquired between different sites and ATP in two complex systems (WT-AK + Asp + ATP + Lys and A380I + Asp + ATP + Lys) for 100 ns dynamic simulation. The numbers of H-bonds between Tyr198 and ATP in mutant A380I were more than that of WT-AK after 20 ns simulation in Figure 6A. The numbers of H-bonds between Arg203 and ATP in mutant A380I were more than that of WT-AK after 20 ns simulation in Figure 6B; A380I can even reach number 5. The numbers of H-bonds of Arg203 were better than in other sites, mostly achieving number 3, which is consistent with Table 2. The numbers of H-bonds between Ser227 and ATP in mutant A380I were significantly more than that of WT-AK after 40 ns simulation in Figure 6C. A380I had more than one H-bond most of the time in Figure 6D. However, WTAK had more than one H-bond only in the first 10 ns. The numbers of H-bonds between Lys228 and ATP in mutant A380I were significantly more than that of WTAK after 20 ns simulation. Therefore, the number of H-bonds in mutants are more than that of WTAK in 100 ns simulation.

**Figure 7:**
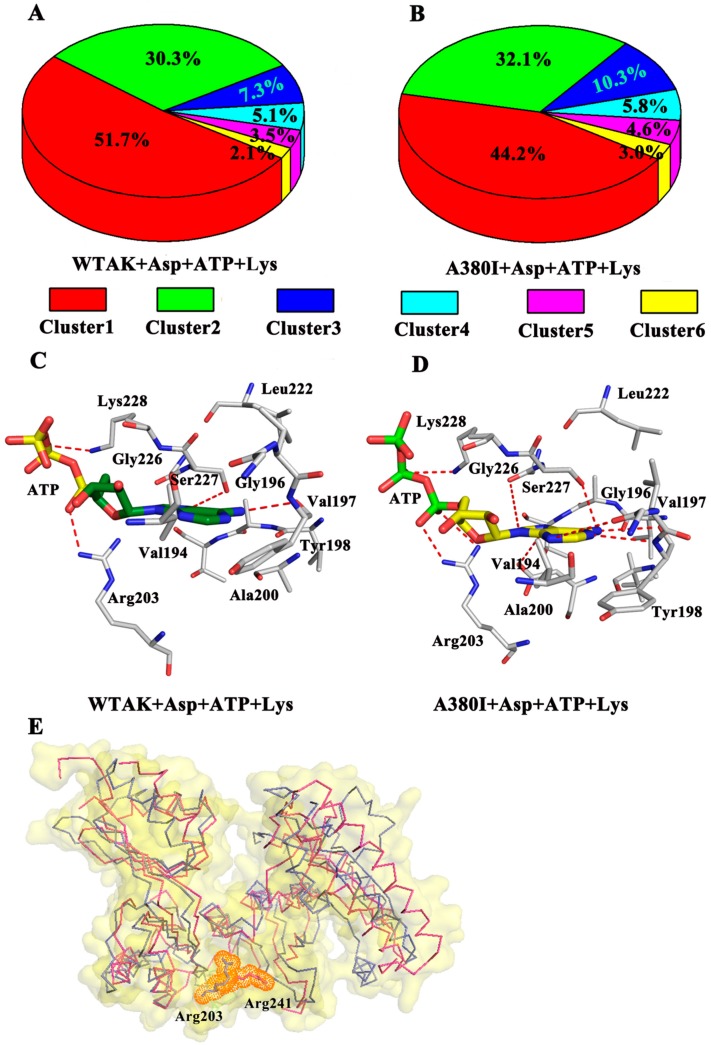
(**A**) Cluster analysis of WT-AK with Asp, ATP and Lys complex. (**B**) Cluster analysis of A380I mutant with Asp, ATP and Lys complex. (**C**) Hydrogen bond network between ATP and WT-AK. (**D**) Hydrogen bond network between ATP and A380I. The red dotted lines in C and D represent hydrogen bonds. (**E**) the pink ribbons represent mjAK and the slate ribbons represent CpAK; slate and pink stick represent residues Arg203 and Arg241 in orange dots.

In addition, cluster analysis was performed on each trajectory of the two systems to reveal a clear-cut structural difference of CpAK and ATP in the different systems (Figure 7). As shown in Figure 7A, the WT-AK + Asp + ATP + Lys complex system was divided into six groups, the percentages of them being 51.7%, 30.3%, 7.3%, 5.1%, 3.5%, and 2.1%, respectively. Similarly, the A380I + Asp + ATP + Lys complex system was also divided into six groups, the percentages being 44.2%, 32.1%, 10.3%, 5.8%, 4.6%, and 3.0%, respectively (Figure 7B). The representative structure of the densest cluster in cluster 1 (represented by Figure 7C,D) was chosen to analyze the interaction between CpAK and ATP in the two systems. The residues Arg203, Ser227 and Lys228 had strong hydrogen bonding with ATP in the representative structure of cluster 1. In the aspartokinase-substrate complex of *Methanococcus jannaschii* AK (mjAK), Arg241 participated in the catalytic reaction [1]. By spatial comparison, site 241 from mjAK corresponded to site 203 from CpAK, which led us to conclude that site 203 plays an important role in the catalytic reaction (Figure 7E). The mutation could lead to enhanced hydrogen bonding between ATP and site 203, thereby enhancing the activity of CpAK.

### 2.5. Mutation Generates an Open Conformation of the Protein

**Figure 8:**
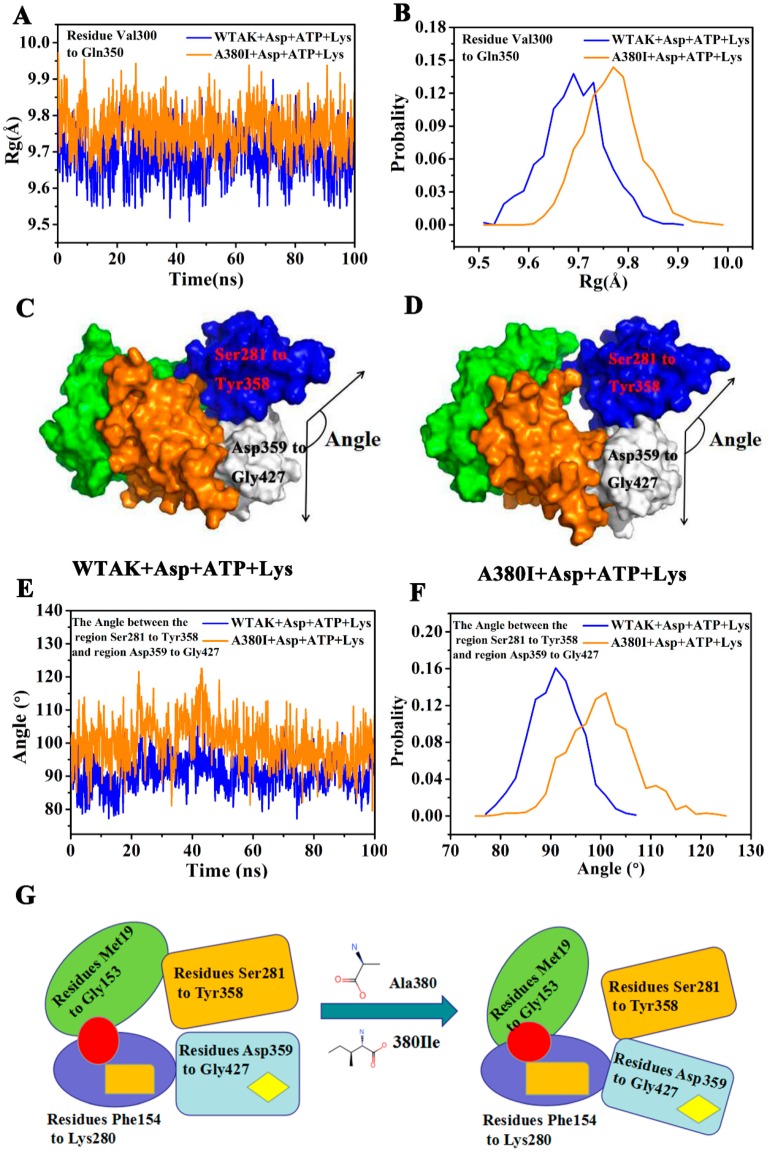
(**A**) Rg plot from residues Val300–Gln350 of the WT-AK and A380I. (**B**) The probability of the Rg of two complexes from residues Val300–Gln350. (**C**) The surface of WT-AK + Asp + ATP + Lys complex (the angle: Cα atom of E394, Cα atom of L315 and Cα atom of V318). (**D**) The surface of A380I + Asp + ATP + Lys complex (the angle: Cα atom of E394, Cα atom of L315 and Cα atom of V318). (**E**) The angle between the region Ser281–Tyr358 and Asp359–Gly427. (**F**) The probability of the angle between the region Ser281–Tyr358 and Asp359–Gly427. (**G**) Schematic models of domain movement for the regulatory mechanism of WT-AK and A380I. Red circle represents the binding site of Asp; orange rectangle represents the binding site of ATP; yellow diamond represents the binding site of Lys.

The Rg value (Figure 8A) and probability (Figure 8B) of the residues Val300–Gln350 were calculated in the 100 ns MD simulation. The Rg of A380I and WT-AK were found to be approximately 9.78 Å and 9.69 Å, respectively. The Rg value of A380I was higher than that of WT-AK, indicating the expansion of residues Val300–Gln350 and the increase of volume caused by the mutation.

The expansion of Val300–Gln350 region resulted in a larger angle between Ser281–Tyr358 and Asp359–Gly427; 100° for A380I mostly and 90° for WT-AK mostly (Figure 8E,F). Protein conformation was open due to the increased angle between Ser281–Tyr358 and Asp359–Gly427 (Figure 8D). This open protein conformation was in a relaxed state (R-state) that facilitated the binding of substrates, thereby affecting the activity of CpAK. This unique feature of mutant strain A380I had not been reported in the literature.

In this study, WT-AK was in the T-state when inhibited by the inhibitor Lys (Figure 8C). After the mutation, conformation of the enzyme became loose (Figure 8D), enzyme activity increased, and feedback inhibition was removed. The mutation increased the hydrogen bond occupancy rate of Arg203 (HN1)-ATP (O). Xiuyun Wu reported that the increased enzyme activity of double mutant S41N/T43E from *Aspergillus niger* XynB was due to the increase of binding ligand molecules from 500 (wild type) to 670, consistent with the conclusion of our current study [28]. In the enzymatic reaction, the phosphoryl groups were provided by ATP, from its active site. The more connection with ATP, the easier it was to catalyze the substrate. Chang-Cheng Li reported that the activity of double mutant D182/R184A of PaAK (*Pseudomonas.uginosa,* Pa) was reduced to 60% due to the double mutation in the ATP binding site, which proved that ATP had a crucial effect on enzyme activity [29]. As can be seen from Figure 8G, the entire conformation of the enzyme was affected by the mutation around the binding site of the inhibitor, which increased the binding of ATP by remote regulation, thereby affecting the enzyme activity.

## 3. Materials and Methods

### 3.1. Experimental Materials

Recombinant *Escherichia coli* (WT-AK) was preserved in our laboratory; plasmid extraction kit, protein electrophoresis marker, nucleic acid electrophoresis marker, and *Dpn*I digestive enzyme were purchased from TaKaRa (Peking, China); Kanamycin and IPTG were purchased from VWR international, LLC (Radnor, PA, USA); Ni Sepharose™ 6 Fast Flow was purchased from General Electric Company (Fairfield, CT, USA); His-Tap (27E8) Mouse mAb (HRP Conjugate) (Art.No.9991S) was purchased from Cell Signaling Technology (Boston, MA, USA).

### 3.2. Test Method

#### 3.2.1. Construction of Strain

The plasmid was extracted from recombinant *E. coli* and was amplified by PCR using mutation primers. The outcome was verified by 1% agarose gel electrophoresis. The methylated template was digested by *Dpn*I enzyme under the conditions: *Dpn*I 0.3 µL, buffer 2 µL, and PCR product 2 µL, at 37 °C for 2 h. The digested products were transferred into competent *E. coli* BL21 cells as follows: The cells were kept in an ice bath for 5 min, at 42 °C for 90 s, and again in an ice bath for 2 min, adding 900 µL LB medium without resistance. The cells were then incubated at 37 °C, 160–170 rpm for 90 min, centrifuged at 8000 rpm for 2 min, and finally 800 µL supernatant was discarded. The residual cells blended were screened on solid plates containing kanamycin. High throughput was further used to screen mutant strains with high enzyme activity; the template was amplified by PCR using cloned primers and products verified by 1% agarose gel electrophoresis, followed by sequencing from Sangon Biotech (Shanghai, China) Co., Ltd.

Mutation primer: 5′----CGCAGAGCTTCCATGAACTCNNNGGTAACACCTG---- 3′

5′----CAGTCTCACCCAGGTGTTACCNNNGAGTTCATGGAAG---- 3′

Cloned primer: 5′----GGAATTCCATATGGCCCTGGTCGTACAGAA----3′

5′----GGAATTCTTAGCGTCCGGTGCCTGCAT----3′

#### 3.2.2. Purification and Validation of Strain

Mutant strains, after successful sequencing, were cultured to an OD_600_ value of 0.6–0.8, after which isopropyl-β-d-thiogalactoside (IPTG) was added at a final concentration of 1 mM to induce growth overnight at 23 °C. The cells were subsequently collected by centrifugation at 4 °C, 8000 rpm for 10 min, and suspended in 10 mL PBS (pH 7.4). A crude enzyme solution, acquired from the supernatant of the second centrifugation (4 °C, 8000 rpm, 10 min), was purified by an Ni-NTA column. The purified solution was verified by SDS-PAGE (12% separation gel and 5% concentration glue) and Western blotting.

Western blotting was performed as follows: The “sandwich” structure, formed by a filter board, PVDF membrane, glue, and another filter board, was put into a semi-humid membrane converter (Bio-Rad, Munich, Germany) with a current of 140 mA for about 1 h. TBST was used to wash the membrane twice for 10 min. The washed membrane was taken out and sealed with 5% skimmed milk powder at room temperature for 1.5 h and then rinsed by TBST. The antibody (2.5 µL), diluted with 3% skim milk powder, was added to it and incubated at 4 °C in a shaking table overnight and rinsed by TBST several times. Finally, the membrane was imaged in the Gel imaging instrument (GE Healthcare Bio-Sciences AB, Fairfield, CT, USA) using ECL to expose the membrane for 30 s a priori.

#### 3.2.3. Determination of Dynamics

The purified enzyme (50 µL) was added to the following reaction system (1 mL): 1.6 mM MgSO_4_, 800 mM KCl, 10 mM L-aspartic acid, 10.4 mM ATP (in 25 mM Tris-HCl), 100 mM Tris-HCl buffer solution (pH 8.0), 10 mM β-mercaptoethanol, and 800 mM NH_4_OH. Different concentrations of L-Asp (0.5–16 mM) were added to the reaction at 28 °C for 30 min, and then an equal volume of FeCl_3_ (FeCl_3_ dissolved in 0.1 M HCl, 12% trichloroacetic acid, and 3 M HCl at a volume ratio of 1:1:1) was added to terminate the reaction, before measuring the absorbance at 540 nm. Water was used instead of L-Asp as a blank control. The nonlinear equation was fitted by Hill equation V = Vmax (Sn)/(Kn + Sn) using Origin 8.5 software (Northampton, MA, USA, Origin Lab).

#### 3.2.4. Determination of Enzymatic Properties

To assess the optimal temperature, 50 µL of purified enzyme was added to the above reaction system, reacting at different temperatures (15, 20, 25, 26, 28, 30, 35, 40, 45, and 50 °C) for 30 min. Water was used instead of L-Asp as a blank control. The highest enzyme activity was defined as 100%. All measurements were repeated thrice.

To assess the optimal pH, 50 µL of purified enzyme was added to reaction systems at different pH (6.0, 6.5, 7.0, 7.5, 8.0, 8.5, 9.0, 9.5, and 10.0), regulated by Tris-HCL.

To assess the stability, 50 µL of purified enzyme was added to the above reaction system at the optimal temperature and pH conditions for 30 min. Enzyme activity was defined as 100%.

To assess the effects of substrate inhibitors on enzyme activities, different inhibitor mixtures (Thr, Lys, Met, Lys + Thr, Lys + Met, Thr + Met, Thr + Lys + Met) were added to the reaction, with each compound at final concentrations of 0.2, 1, 5, or 10 mM. The reaction was carried out in 96-well plates.

#### 3.2.5. Molecular Dynamics Simulation

Using GROMOS 53A6 force field to describe proteins and ligands, the parameters of ligands were provided by the PRODRG2.5 server [24,30]. All complex systems were modeled by MD simulation in the periodic boundary frame of SPC water model [31], in which chloride and sodium ions were added to neutralize the system. Energy minimization was achieved through the steepest descent method to generate the balanced initial structure. Subsequently, the system was maintained in a stable environment (300 K, 1 bar) using 100 ps NVT (Berendsen temperature coupled with constant particle number, volume, and temperature) and 100 ps NPT (Parrinello-Rahman pressure coupled with fixed particle number, pressure, and temperature) [32]. The coupling constants of temperature and pressure were set at 0.1 and 2 ps respectively. The particle mesh Ewald algorithm was used to describe the long-range electrostatic interaction whose order was four, grid spacing was 1.6 Å, and Van der Waals interaction was calculated at the cut-off value of 14 Å [33]. All key lengths were constrained by the LINCS algorithm [34]. After the thermodynamic properties were stabilized, the 100 ns molecular system was simulated with 2-fs step size, and the coordinates of all models were saved every 2 ps.

## 4. Conclusions

A mutant A380I was obtained by high-throughput screening of a novel AK monomer from *Corynebacterium pekinense*, whose enzyme activity was increased 11.32-fold after mutation. Results showed that the optimal temperature was increased by 2 °C, optimal pH was the same as that of WT-AK, and the stability was extended to 6.0 h. The inhibition of A380I was weakened at various inhibitor concentrations and activated at certain inhibitor concentrations (10 mM Lys, 5 mM or 10 mM Lys + Thr, 10 mM Lys + Met, 5 mM Lys + Thr + Met).

In order to investigate the mechanism of the increase of enzyme activity and the release of feedback inhibition after mutation, two complex systems (WT-AK + Asp + ATP + Lys and A380I + Asp + ATP + Lys) were selected for 100 ns MD simulations. The effect of inhibitors (Lys) on the mutant A380I were reduced. The mutant A380I enhanced the RMSD values of residues at the binding pocket of Asp (Val120–Gly170) and ATP (Ieu220–Glu270), indicating a stronger interaction with Asp and ATP (Figure 5). In fact, Arg203, Ser227 and Lys228 had strong hydrogen bonding with CpAK. Upon comparison, we concluded that Arg203 played an important role in the catalytic reaction and A380I enhanced the occupancy rate of hydrogen bonding in Arg203 (HN1)-ATP (O).

The angle between Ser281–Tyr358 and Asp359–Gly427 increased, resulting in an open conformation (Figure 8D), which corresponds to a relaxed state (R-state). It facilitated the binding of substrates, thus affecting the activity of CpAK. These findings are of great significance for the improved yield of related amino acids by restructuring AK to relieve feedback inhibition [35].

## Figures and Tables

**Table 1:** Substrate inhibitor of WT-AK and A380I.

Enzyme	WT-AK				A380I			
Inhibitors	Relative Activity (%) Concentration (mM)							
	0.2	1	5	10	0.2	1	5	10
Control	100	100	100	100	100	100	100	100
Lys	93.87 ± 1.54	91.13 ± 2.69	88.33 ± 1.77	85.43 ± 1.52	94.91 ± 0.66	95.47 ± 0.69	96.27 ± 0.96	112.41 ± 1.18
Thr	95.02 ± 2.65	94.42 ± 0.45	89.37 ± 0.91	89.76 ± 2.90	84.49 ± 1.66	94.61 ± 0.83	95.48 ± 2.55	97.92 ± 1.04
Met	92.01 ± 2.40	83.11 ± 1.46	72.66 ± 0.88	73.7 2± 1.65	90.57 ± 0.82	95.73 ± 0.96	97.82 ± 0.78	98.35 ± 0.79
Lys + Thr	71.01 ± 3.65	59.26 ± 2.35	40.02 ± 1.45	34.77 ± 0.97	90.04 ± 0.94	96.94 ± 1.46	102.07 ± 3.12	104.48 ± 0.67
Lys + Met	80.31 ± 0.84	75.67 ± 1.31	72.97 ± 0.68	69.65 ± 1.56	90.51 ± 1.05	93.29 ± 2.18	99.12 ± 2.13	104.50 ± 1.36
Thr + Met	84.76 ± 1.59	81.66 ± 0.88	78.86 ± 1.35	70.91 ± 1.00	71.87 ± 2.02	85.19 ± 2.75	94.07 ± 0.091	97.46 ± 1.85
Lys + Thr + Met	76.51 ± 1.03	60.06 ± 1.88	49.16 ± 2.61	30.51 ± 0.78	84.17 ± 1.25	99.73 ± 1.95	105.01 ± 1.53	99.79 ± 2.13

The underline indicates activation

**Table 2:** Hydrogen bond occupancy between ATP and CpAK during 100 ns MD.

Hydrogen Bonds Occupancies		WTAK	A380I
Donor	Accepter		
Lys228:NZ	ATP:O3	47.49%	56.26%
Arg203:NH1	ATP:O	38.34%	51.78%
Arg203:NH1	ATP:O1	43.11%	49.55%
Arg203:NH2	ATP:P	44.01%	63.27%
Arg203:NH2	ATP:O1	48.00%	57.76%
Lys228:NZ	ATP:O1	34.33%	45.57%
Arg203:NH2	ATP:O9	90.62%	93.89%
Ser227:OG	ATP:N5		32.53%
ATP:N5	Tyr198:O	42.48%	54.35%
ATP:N5	Ser227:OG	31.74%	39.14%
ATP:N5	Ala200:O	27.52%	32.84%
Tyr198:N	ATP:N4	55.79%	55.79%
Ser281:OG	ATP:O3	28.14%	41.37%
ATP:C1	GLY226:O		45.38%
Val194:CG1	ATP:O11		34.21%
Tyr198:CD1	ATP:C10		31.97%

## References

[1] Faehnle C.R., Liu X.Y., Pavlovsky A., Viola R.E. (2006). The initial step in the archaeal aspartate biosynthetic pathway catalyzed by a monofunctional aspartokinase. Acta Cryst..

[2] Chen Z., Rappert S., Sun J.B., Zeng A.P. (2011). Integrating molecular dynamics and co-evolutionary analysis for reliable target prediction and deregulation of the allosteric inhibition of aspartokinase for amino acid production. J. Biotechnol..

[3] Min W.H., Li H.Y., Li H.M., Liu C.L., Liu J.S. (2015). Characterization of Aspartate Kinase from Corynebacterium pekinense and the Critical Site of Arg169. Int. J. Mol. Sci..

[4] Dong X.Y., Zhao Y., Zhao J.X., Wang X.Y. (2016). Characterization of aspartate kinase and homoserine dehydrogenase from Corynebacterium glutamicum IWJ001 and systematic investigation of l-isoleucine biosynthesis. J. Ind. Microbiol. Biotechnol..

[5] Galili G. (1995). Regulation of Lysine and Threonine Synthesis. Plant cell.

[6] Richaud C., Mazat J.P., Felenbok B., Patte J.C. (1974). The role of lysine and leucine binding on the catalytical and structural properties of aspartokinase III of *Escherichia coli* K. 12. Eur. J. Biochem..

[7] Curien G., Laurencin M., Robert-Genthon M., Dumas R. (2007). Allosteric monofunctional aspartate kinases from Arabidopsis. FEBS J..

[8] Paris S., Viemon C., Curien G., Dumas R. (2003). Mechanism of control of Arabidopsis thaliana aspartate kinase-homoserine dehydrogenase by threonine. J. Biol. Chem.

[9] Curien G., Ravanel S., Robert M., Dumas R. (2005). Identification of six novel allosteric effectors of Arabidopsis thaliana aspartate kinase-homoserine dehydrogenase isoforms. Physiological context sets the specificity. J. Biol. Chem..

[10] Angeles T.S., Viola R.E. (1990). The kinetic mechanisms of the bifunctional enzyme aspartokinase-homoserine dehydrogenase I from *Escherichia coli*. Arch. Biochem. Biophys..

[11] Veron M., Guillou Y., Cohen G.N. (1985). Isolation of the aspartokinase domain of bifunctional aspartokinase I-homoserine dehydrogenase I. from *E.coli* K12. FEBS Lett..

[12] Dong X., Quinn P.J., Wang X. (2011). Metabolic engineering of Escherichia coli and Corynebacterium glutamicum for the production of l-threonine. Biotechnol. Adv..

[13] Yoshida A., Tomita T., Kurihara T., Fushinobu S., Kuzuyama T., Nishiyama M. (2007). Structural Insight into concerted inhibition of α2β2-type aspartate kinase from *Corynebacterium glutamicum*. J. Mol. Biol..

[14] Kalinowski J., Cremer J., Bachmann B., Eggeling L., Sahm H., Pühler A. (2010). Genetic and biochemical analysis of the aspartokinase from *Corynebacterium glutamicum*. Mol. Microbiol..

[15] Muehlbauer G.J., Somers D.A., Matthews B.F., Gengenbach B.G. (1994). Molecular genetics of the maize (Zea mays L.) aspartate kinase-homoserine dehydrogenase gene family. Plant. Physiol..

[16] Kotaka M., Ren J., Lockyer M., Hawkins A.R., Stammers D.K. (2006). Structures of R- and T-state Escherichia coli aspartokinase III Mechanisms of the allosteric transition and inhibition by lysine. J. Biol. Chem..

[17] Schuldt L., Suchowersky R., Veith K., Mueller-Dieckmann J., Weiss M.S. (2011). Cloning, expression, purification, crystallization and preliminary X-ray diffraction analysis of the regulatory domain of aspartokinase (Rv3709c) from *Mycobacterium tuberculosis*. Acta Crystallogr. Sect. F Struct. Biol. Cryst. Commun..

[18] Chen Z., Meyer W.Q., Rappert S., Sun J.B., Zeng A.P. (2011). Coevolutionary Analysis Enabled Rational Deregulation of Allosteric Enzyme Inhibition in Corynebacterium glutamicum for Lysine Production. Appl. Environ. Microbiol..

[19] Robin A.Y., Cobessi D., Curien G., Robert-Genthon M., Ferrer J.L., Dumas R. (2010). A New Mode of Dimerization of Allosteric Enzymes with ACT Domains Revealed by the Crystal Structure of the Aspartate Kinase from *Cyanobacteria*. J. Mol. Biol..

[20] Manjasetty B.A., Chance M.R., Burley S.K., Panjikar S., Almo S.C. (2014). Crystal structure of Clostridium acetobutylicum Aspartate kinase (CaAK): An important allosteric enzyme for amino acids production. Biotechnol. Rep..

[21] Han G., Hu X., Qin T., Li Y., Wang X. (2016). Metabolic engineering of *Corynebacterium glutamicum* ATCC13032 to produce S-adenosyl-l-methionine. Enzym. Microb. Technol..

[22] Jiang X.K., Chen G.J., Wang L.S. (2016). Structural and dynamic evolution of the amphipathic *N*-terminus diversifies enzyme thermostability in the glycoside hydrolase family 12. Phys. Chem. Chem. Phys..

[23] Eisold A., Labudde D. (2018). Detailed Analysis of 17β-Estradiol-Aptamer Interactions: A Molecular Dynamics Simulation Study. Molecules.

[24] Guan S.S., Zhao L., Jin H.Y., Shan N., Han W.W., Wang S., Shan Y.M. (2017). Binding modes of phosphotriesterase-like lactonase complexed with -nonanoic lactone and paraoxon using molecular dynamics simulations. J. Biomol. Struct. Dyn..

[25] Zheng F., Tu T., Wang X.Y., Wang Y., Ma R., Su X.Y., Xie X.M., Yao B., Luo H.Y. (2018). Enhancing the catalytic activity of a novel GH5 cellulase GtCel5 from Gloeophyllum trabeum CBS 900.73 by site-directed mutagenesis on loop 6. Biotechnol. Biofuels.

[26] Yoshida A., Tomita T., Kuzuyama T., Nishiyama M. (2010). Mechanism of concerted inhibition of α2β2-type hetero-oligomeric aspartate kinase from *Corynebacterium glutamicum*. J. Biol. Chem..

[27] Thongekkaew J., Ikeda H., Masaki K., Iefuji H. (2013). Fusion of cellulose binding domain from Trichoderma reesei CBHI to Cryptococcus sp S-2 cellulase enhances its binding affinity and its cellulolytic activity to insoluble cellulosic substrates. Enzym. Microb. Technol..

[28] Wu X.Y., Tian Z.N., Jiang X.K., Zhang Q., Wang L.S. (2018). Enhancement in catalytic activity of Aspergillus niger XynB by selective site-directed mutagenesis of active site amino acids. Appl. Microbiol. Biotechnol..

[29] Li C.C., Yang M.J., Liu L., Li T., Peng C.T., He L.H., Song Y.J., Zhu Y.B., Shen Y.L., Yang J. (2018). Mechanistic insights into the allosteric regulation of *Pseudomonas* aeruginosa aspartate kinase. Biochem. J..

[30] Schuttelkopf A.W., van Aalten D.M. (2004). PRODRG: A tool for high-throughput crystallography of protein-ligand complexes. Acta Crystallogr. D Biol. Crystallogr..

[31] Hess B., van der Vegt N.F.A. (2006). Hydration thermodynamic properties of amino acid analogues: A systematic comparison of biomolecular force fields and water models. J. Phys. Chem. B.

[32] Berendsen H.J.C., Postma J.P.M., van Gunsteren W.F., DiNola A., Haak J.R. (1984). Molecular dynamics with coupling to an external bath. J. Chem. Phys..

[33] Parrinello M., Rahman A. (1981). Polymorphic transitions in single-crystals: A new molecular-dynamics method. J. Appl. Phys..

[34] Hess B.B., Bekker H., Berendsen H.J.C., Fraaije J.G.E.M. (1997). LINCS: A linear constraint solver for molecular simulations. J. Comput. Chem..

[35] Wang J., Gao D., Yu X., Li W., Qi Q. (2015). Evolution of a chimeric aspartate kinase for L-lysine production using a synthetic RNA device. Appl. Microbiol. Biotechnol..

